# Researchers' perspectives on public involvement in health research in Singapore: The argument for a community‐based approach

**DOI:** 10.1111/hex.12915

**Published:** 2019-07-19

**Authors:** Lidia Luna Puerta, Bernadette Bartlam, Helen E. Smith

**Affiliations:** ^1^ Family Medicine and Primary Care, Lee Kong Chian School of Medicine Nanyang Technological University Singapore Singapore Singapore; ^2^ Research Institute for Primary Care & Health Sciences Keele University Keele UK; ^3^ Division of Public Health and Primary Care Brighton and Sussex Medical School Brighton UK

**Keywords:** Asia, attitudes, cultural contexts, health research, public involvement, qualitative methods, qualitative research, research design, research personnel, Singapore

## Abstract

**Background:**

Singapore is becoming a world‐class research hub, promoting the advancement of patient care through translational clinical research. Despite growing evidence internationally of the positive impact of public involvement (PPI), in Singapore PPI remains unusual beyond patient participation as subjects in studies.

**Objective:**

To explore health researchers' understandings of the principles, role and scope of PPI, and to identify barriers and opportunities for implementation in Singapore.

**Design:**

Semi‐structured qualitative interviews between April and July 2018. Data were analysed using thematic framework analysis.

**Results:**

Whilst most participants (n = 20) expressed a lack of experience of PPI, the interview process provided an opportunity for reflection through which it emerged as a beneficial strategy. Interviewees highlighted both utilitarian and ethical reasons for implementing PPI, particularly around increasing the relevance and efficiency of research. In addition to those challenges to PPI documented in the existing literature, participants highlighted others specific to the Singaporean context that make PPI at an individual level unlikely to be successful, including the socio‐political environment and prevailing social and professional hierarchies. They also identified asset‐based strategies to overcome these, in particular, a more community‐oriented approach.

**Conclusion:**

The cultural reluctance of individuals to question perceived authority figures such as researchers may be overcome by adopting an approach to PPI that is closer to family and local community values, and which facilitates patients and the public collectively engaging in research. Further work is needed to explore the views of patients and the public in Singapore, and the implications for other Asian communities.

## 1 INTRODUCTION

### 1.1 Background on public involvement

There is growing evidence that public involvement (PPI) in research, defined as research being carried out “with” or “by” members of the public rather than “to,” “about” or “for” them,^1^ carries a number of benefits, including the production of higher quality, more efficient and effective research.^2^, ^3^, ^4^, ^5^, ^6^, ^7^, ^8^, ^9^, ^10^, ^11^, ^12^, ^13^, ^14^, ^15^, ^16^ Such evidence has resulted in some health research funders, such as the UK's National Institute for Health Research (NIHR)^2^ making it a mandatory consideration involving human participation. Although patient involvement can be interpreted in various ways, the key principle is of active PPI in the activities, organization and governance of health research, whether in specific projects or research more generally.^2^

Arguably, the case for PPI is rooted in the principles of biomedical ethics. The utilitarian case argues that by incorporating public knowledge and expertise, researchers can identify user‐centred research objectives and questions; develop more appropriate research materials; enhance recruitment strategies; enrich data analysis; improve cost‐effectiveness^10^, ^17^, ^18^; and optimize dissemination, implementation and impact of the research findings.^6^, ^10^, ^11^, ^13^, ^19^, ^20^ As a result, PPI is being embraced not only by many public bodies but by commercial enterprises, such as the pharmaceutical industry.^17^, ^18^, ^21^

Balancing this utilitarian model is the Kantian “Categorical Imperative” arguing that human beings should not be treated as a means to an end.^22^, ^23^ From this perspective, it is seen that members of the public are legitimate, central stakeholders in research affecting their health and that of their communities. PPI emphasizes the importance and role of lay knowledge. It promotes patients and members of the public as experts in their “lived experience” both entitled to be, and having a responsibility to be, meaningful partners rather than passive subjects and/or recipients of research.^24^, ^25^, ^26^, ^27^ In doing so, it, first, challenges the authority of traditional expert knowledge and, second, plays a crucial role in opening up research evidence to public scrutiny.

### 1.2 The Singaporean context

Whilst Western models have tended to emphasize the contribution of individuals,^28^, ^29^ there is a growing trend to move to PPI strategies that are more inclusive and engage those who are most disadvantaged, with the greatest health needs. So far as we are aware, there is little consideration of the concept of PPI or its use in Asian countries. Singapore provides an interesting case study for exploring the potential of PPI within an Asian context.

Since becoming an independent nation in 1965, Singapore has experienced dramatic economic and social change. In little over 60 years, it has risen from “Third World” poverty^30^ to being an “Asian Tiger,” sitting alongside Hong Kong, South Korea and Taiwan.^31^ The drive to survive that powered this enormous transition is now focused on addressing the tension between its political and cultural position as an outward facing country and its geographical context, surrounded by traditional Asian cultures with a growing emphasis on religious fundamentalism, for example Indonesia and the Philippines.^32^ Given these existential challenges, it is perhaps not surprising that Singaporean culture privileges the collective rather than the individual experience.^33^ As an island city‐state with scarce geographical resources, a resident population that has doubled in the past 50 years^34^ and which is one of the most rapidly ageing in the world,^35^ the Government has prioritized investment in knowledge capital.^36^, ^37^ In 2016, SG$19 billion (US$14 billion) was invested in education and research.^38^ Consequently, Singapore is rapidly becoming a world‐class research hub, attracting globally renowned scholars and researchers.^38^, ^39^ Whilst this drive to innovation and excellence in research includes an emphasis on the advancement of patient care through translational clinical research,^40^ PPI is unusual. This paper reports findings from interviews with researchers in Singapore, exploring their experiences and views of PPI.

### 1.3 Study aims

To explore:

- The extent to which those working in health research understand the principles, role and scope for involving patients and the public in health research.
- The challenges and opportunities for implementing PPI in Singapore.

## 2 METHODS

An exploratory, qualitative design was used of face‐to‐face semi‐structured interviews. These were designed to offer the opportunity for participants to reflect on their experiences and produce their own narratives, being guided rather than lead by the interviewer.^41^, ^42^ The initial topic guide was informed by the literature on the scope and practice of PPI. It was adapted iteratively as the interviews proceeded to take account of emerging themes to be explored in subsequent interviews. These emerging themes were identified through the use of reflexive notes (see below) and preliminary data analysis. Brief personal profile data on each individual were collected in order to contextualize accounts and experiences (Table 1). All interviews were conducted by LLP.

**Table 1:** Participant characteristics

	Gender	Ethnic group	Position	Research experience (y)	Research field
R01	Female	Chinese	Research Fellow	6‐10	Pregnancy & Parenting
R02	Male	Caucasian	Doctoral Researcher	1‐5	Older adults
R03	Female	Chinese	Doctoral Researcher	1‐5	Neurology
R04	Female	Caucasian	Doctoral Researcher	1‐5	Pregnancy & Parenting
R05	Male	Chinese	GP Researcher	<1	Family Medicine
R06	Male	Indian	Assistant Professor	>10	Chronic conditions
R07	Male	Chinese	Doctoral Researcher	1‐5	Psychiatry
R08	Female	Caucasian	Associate Professor	>10	Chronic conditions
R09	Male	Chinese	GP Researcher	1‐5	Family Medicine
R10	Male	Chinese	Research Manager	>10	General population
R11	Male	Chinese	Assistant Professor	>10	Older adults
R12	Female	Indian	Adjunct Associate Professor	>10	Psychiatry
R13	Female	Chinese	Research Manager	<1	General population
R14	Male	Chinese	Associate Professor	>10	Physiology of ageing
R15	Female	Chinese	Professor	>10	Neurology
R16	Female	Chinese	Professor	>10	Older adults
R17	Female	Chinese	Doctoral Researcher	<1	Chronic conditions
R18	Female	Chinese	Occupational Therapist	6‐10	Physiology of ageing
R19	Male	Malay	Assistant Professor	>10	Chronic conditions
R20	Female	Chinese	Associate Professor	>10	Patients with cancer

### 2.1 Recruitment

Potential participants were identified through purposive sampling from searching the websites of different research institutions in Singapore and by snowball sampling.^43^ The intention was to include informant rich cases to ensure a wide range of perspectives so as to learn as much as possible about the topic under investigation (Table 1). Invites were sent via email with a study flyer attached. When people responded positively, an information sheet and consent form were sent, together with arrangements for the date, time and location of the interview. Participants were eligible for inclusion if they were currently involved in health‐related research in Singapore and were able to communicate in English. All participants received a SGD15 voucher as a token of appreciation. Data collection took place between April and July 2018, and all interviews were conducted at the workplace of participants.

Informed consent was obtained at the beginning of the interview and confirmed again at the end. All interviews were audio‐recorded, transcribed verbatim, cleaned and anonymized before analysis. Participants were assigned a code in replacement of their identity, consisting of the letter “R” followed by a two‐digit number in order of sequential recruitment (ie R01), gender, ethnicity, position and years of experience. All data were stored securely in line with Nanyang Technological University's requirements for safe data storage.^44^ Data saturation was based on inductive thematic saturation (see below), and recruitment ceased when no new themes were emerging.^45^

### 2.2 Analysis

**Figure 1:**
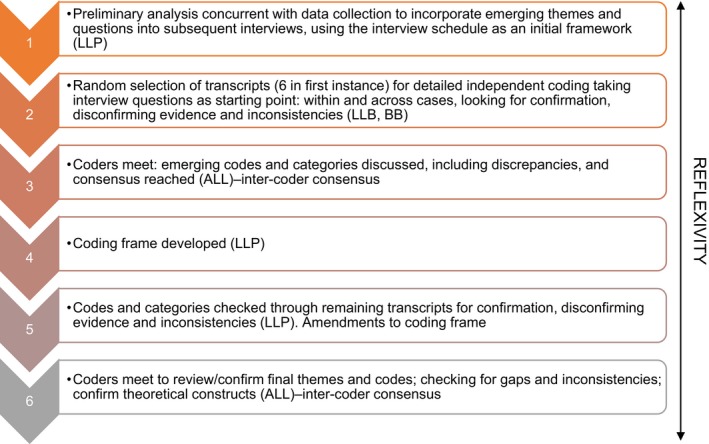
The six stages of coding

Socio‐demographic and professional data were summarized descriptively. A six‐stage analysis plan was agreed based on thematic analysis^46^, ^47^ (Figure 1). To ensure reliability, a process of inter‐coder consensus^48^ was adopted, two authors (LLP and BB) independently coded six randomly selected transcripts and developed a coding frame which was then applied to the remaining data by LLP. Where there were discrepancies, the third author also read the data to reach consensus. This coding frame was further revised as codes were dropped and/or emerged as more dominant. The analysis process was both iterative and concurrent with data collection,^49^ increasingly moving from the concrete reality of data to abstract, theoretical constructions.^50^

### 2.3 Reflexivity

Detailed reflexive notes were made about each interview as soon as possible post‐interview. These contact summary forms included notes on unexpected themes that arose might warrant reviewing in subsequent interviews.^51^ The process of reflexivity continued during the analysis in the form of memos (Figure 1).

### 2.4 Ethics

The study was reviewed and approved by Nanyang Technical University's Internal Review Board, April 2018 (ref: IRB‐2018‐02‐028).

## 3 RESULTS

A total of 36 potential participants were contacted: 26 (72%) replied and 20 (56%) were interviewed (Table S1 provides the reasons six potential participants were not interviewed). The average interview lasted approximately 1 hour. The total amount of audio data was 22 and a quarter hours.

### 3.1 Participants

Participants were placed in four categories of experience: <1; 1‐5; 6‐10; and >10 years. The majority (n = 12) had 6 years or more experience. Five people were doctoral students; four of whom were practising clinicians with previous experience of research; nine were working at professorial level; and two were involved in research administration, including recruitment. Reflecting the ethnicity of Singapore, 14 were Chinese, two were Indian, one was Malay, with three Caucasians. Research backgrounds were diverse, including five people working with older adults and four with patients with chronic diseases. There was an even gender split, with just over half of participants women (n = 11; Table 1).

Three major themes emerged: reasons to adopt PPI, adoption challenges and opportunities for implementation. In what follows, we present details of these themes using illustrative quotations, before turning to look at the implications. Unique identifiers include participant number, gender, ethnicity, role and length of research experience.

### 3.2 Reasons for adoption

Interviewees identified a number of utilitarian reasons for including PPI, of which the most frequently mentioned was making their research “easier” by either optimizing the design, and/or enhancing recruitment:

They [the public] could actually help the research to be more efficient, to recruit much easier if they're involved. (R15, F, Chinese, Professor, >10)

Public involvement was also seen as helpful in developing strategies to support longer‐term participation, with clear implications for the feasibility of the study in later stages “because they [the research team] can understand the public better… to … bring these people, participants, back, again and again, year after year to continue the study” (R09, M, Chinese, GP Researcher, 1‐5). Part of such efficiency was the potential for PPI to maximize the efficient use of resources, not least financially: “And [PPI would] probably save a lot of money for the grant bodies. (R06, M, Indian, Assistant Professor; >10).”

Public involvement was also seen as an advantage in attracting funding: “Because if we are talking about translational research and that's your area of study, showing that your study is centred around your patient is the number one criteria” (R14, M, Chinese, Associate Professor, >10). This was seen as of particular importance in longitudinal studies, where ensuring sustainability over time is critical: “I think it would be definitely more sustainable. We would probably get more support from everybody else. And even probably better, more sustainable funding” (R09, M, Chinese, GP Researcher, 1‐5). For one researcher, community engagement was essential in involving the public in research. When asked to define PPI, she said “Oh, you mean ground community ambassadors” and went on to describe how such members of the public “could be bringing their community, their family, their friends, their cousins [to the research]” (R13, F, Chinese, Research Manager, <1). She emphasized the importance of identifying key community stakeholders, giving an instance of one person who “she's a nurse herself, and she has many communities, church and all, so she is going to help me to spread the word” (R13, F, Chinese, Research Manager, <1). Working in this way offered a means for building the relationship needed “to pass on the message to a wider group than what the researchers and scientists can” (R09, M, Chinese, GP Researcher, 1‐5). Such an approach fits within the wider shift in Singapore where policy is requiring a change in emphasis from hospital to community‐based health services: “It just comes along as part of the whole landscape […] putting it back into the community is always good (R10, M, Chinese, Research Manager, >10).”

Having a public perspective from the earliest stages was also seen as a way to avoid potentially costly and avoidable mistakes:

If I don't do this [PPI] it might also lead to a lot more problems later … you know, you do it very quickly and then you come up with a design that is not good. Then you actually have to do a lot of patchwork to try and solve the problems. (R16, F, Chinese, Professor, >10)

Public involvement perspectives were also seen as complementary to researchers' knowledge:

Because researchers themselves, you don't know everything. You don't know what's on the ground level; the daily operations, challenges and things like that. (R18, F, Chinese, Occupational Therapist, 6‐10)

It could also bridge the potential disconnect between researchers and the public:

In my view, it is a mistake that we just look at the evidence…sometimes, as researchers… you no longer hear right what people are saying… We think we know because we read what our peers publish in the journals, but do we really know?. (R08, F, Caucasian, Associate Professor, >10)

Linked to this, aligning research to patient and public priorities was identified as potentially allowing a better understanding of needs and expectations: “We want to make sure that whatever we are designing or whatever we are coming up with needs to, first of all, meet the needs of patients, and needs to be something they want. So, I think for sure, in clinical and health services research, patient and PPI is critical, is important and critical” (R20, F, Chinese, Associate Professor, >10). Some participants recognized the need for an inclusive approach that prioritizes the needs of patients:

So there is room for the top‐down approach, but then at the same time we need another set of research coming up, participator from bottom‐up. To say: ok, yes it is strategic, but if you want programs that are going to be effective, we need to engage these real people to tell us what's going to be effective for this cohort, for this group. (R18, F, Chinese, Occupational Therapist, 6‐10)

Interviewees also saw PPI as a way of increasing impact and ensuring that research findings can be translated into practice:

I think it will be more helpful because you have one additional perspective that will help you, later on, to translate it much easier than if you didn't have it. (R15, F, Chinese, Professor, >10)

### 3.3 Adoption challenges

The overarching challenge to the adoption of PPI was a lack of understanding of its role and function particularly on the part of some who had the least experience. This resulted in uncertainty as to how best to operationalize it: “If I was gonna organise it by myself for future studies I wouldn't know how to go about it” (R02, M, Caucasian, Doctoral Researcher, 1‐5). The lack of understanding was reflected in perceptions of the status of patients in research, often seeing them as a means to an end, rather than collaborators, highlighting an imbalance of power: “Absolutely not necessary… it's your paper, not their paper… They are subjects” (R05, M, Chinese, GP Researcher, <1). More experienced participants, themselves supportive of PPI, also identified the lack of understanding as a challenge. They saw this particularly in extent to which researchers might have confidence in the contribution of individuals:

At the back of their minds would be whether or not this member of the public has enough knowledge or sophistication, or understanding, to be able to give you useful input, rather than just say anything they like. (R10, M, Chinese, Research Manager, >10)

Alongside this, researchers identified current practices that treat participants as passive subjects as undermining active engagement and the principles of PPI: “In taking part in research they feel very much like experiments or they feel like a laboratory rat or a guinea pig… that disempowers them” (R03, F, Chinese, Doctoral Researcher, 1‐5). Researchers with more experience also spoke of patients themselves seeing their role as being unequal and of lesser value: “When they hear that we want them to come in and be interviewed, they feel a bit overwhelmed because they feel ‘I am not that educated’ or ‘who am I to talk?’.” (R12, F, Indian, Associate Professor, >10). Consequently, if they asked members of the public to be involved in their research, people would turn down the opportunity because of feeling “incapacitated to offer insight” (R08, F, Chinese, Associate Professor, >10).

This power imbalance within research was reflected in cultural norms that could further hinder collaboration:

It's just cultural, I think it's like that student‐teacher, doctor‐patient type hierarchy relationship. So, a student will never argue with their teacher. So, the teacher can never be wrong. In the same way, the doctor can never be wrong. Whereas, I think in jurisdictions maybe outside Asia…it's quite common for patients to verbalise what they feel […] Because of the culture [in Singapore] patients just don't speak up about what they need… [they] rarely question doctors. (R06, M, Indian, Assistant Professor, >10)

Linked to this, the broader socio‐political context of Singapore was also seen as a potential challenge to patient autonomy, and consequently individual collaboration: “I think it probably has to do with Singapore still being a more traditional paternalistic society (R04, F, Caucasian, Doctoral Researcher, 1‐5).”

Such tensions around the level of contribution and collaboration were also reflected in a lack of clarity around the ethical dimensions of PPI:

I think that there is a great barrier in terms of ethics. It's because we are very used to the traditional forms of ethics application where participants are involved in a certain research project and then there are boundaries as to what they do and what they do not do. So, I don't know whether giving ideas to the researcher on the research, whether it is within the boundary or outside of the boundary. I really don't know. I don't know where it would come under, in terms of ethics. (R02, M, Caucasian, Doctoral Researcher, 1‐5)

Finally, time and resources also emerged as a potential challenge, particularly for those lacking experience in how to effectively incorporate PPI into their work:

I have time limits as to how long I can involve in my project. So that makes it very difficult. There is also cost of involvement, because… I would very much want to involve feedback. But it was not possible; it was the time limits, and also the cost. I did not factor in the cost. So, it's very hard. (R01, F, Chinese, Research Fellow, 6‐10)

### 3.4 Opportunities for implementation

Interviewees highlighted the concept of community‐based PPI that would require outreach activities as a way of “being closer to the community” (R03, F, Chinese, Doctoral Researcher, 1‐5), and introducing research in wider “talk about citizenship [and] be creative […] so that this information does get passed onto them” (R11, M, Chinese, Assistant Professor, >10). Secondly, interviewees highlighted the potential of “respectable people in the community” (R09, M, Chinese, GP Researcher, 1‐5) to pass on messages to the public and act as role models and advocates for PPI. Thirdly, researchers could use “ground community ambassadors” (R13, F, Chinese, Research Manager, <1) “to go out to the community to promote [PPI]” (R15, F, Chinese, Professor, >10), as a way of encouraging involvement. This would result in groups of PPI pioneers, and PPI turning “into a little community thing” (R13, F, Chinese, Research Manager, <1). Finally, an interviewee noted that it would be important to “send it [ideas for implementation of PPI] back to the community” (R11, M, Chinese, Assistant Professor, >10) before developing initiatives.

Building trust between the researchers and communities was seen as key to the successful implementation of PPI: “You have to earn trust first. You have to treat people as people first. To win trust first, to explain what you are doing, and to explain what you don't know about the research; and how they are contributing” (R20, F, Chinese, Associate Professor, >10). Part of this was around integrating the principles of PPI into Singaporean culture itself:

It's almost like inculcating, developing, nurturing this kind of idea to be part of the culture. So, it's not just educating, it's like how do you make it part of your lifestyle?. (R15, F, Chinese, Professor, >10)

With this in mind, participants highlighted the need to raise awareness of research amongst all ages and different ethnic groups, starting with young people:

I would say the best way may be to start doing it is in schools, maybe in young children, maybe in teenagers […] to let them know what does a research process involve, and what is good research, and how can the public influence scientists and researchers, and coming out with research directions and topics. (R09, M, Chinese, GP Researcher, 1‐5)

Interviewees also saw the current emphasis on lifelong education in Singapore as an opportunity to educate older adults on the principles of PPI:

We do have SkillsFuture in Singapore right now going on, so you could have a continuing education module on that [on research and PPI]. (R15, F, Chinese, Professor, >10)

Strategic leadership was considered key to successful implementation, with interviewees identifying the need for policymakers to reward PPI: “I think what we reward and what we don't reward is important. So, you need to reward the right behaviour, you need to reward the right design and so on…To put it as a requirement that your research has got to involve this kind of people. It will help to bring it to be more conscious for people to focus more around the patient”. (R14, M, Chinese, Associate Professor, >10). In order to meet such a requirement on the part of the funders, education and awareness raising amongst professionals were seen to be crucial: “I think [implementing PPI] through educating doctors [what it is] actually about… because I do think people would generally feel it is a good idea” (R02, M, Caucasian, Doctoral Researcher, 1‐5).

Interviewees identified such education occurring at two levels. Firstly, working with emerging researchers and health professionals so that the theories and concepts of PPI are normalized: “When you teach research, it would be one of the things that you can consider, especially undergraduate education, all this. So, when you introduce research you can bring this topic in. And after that, they would like to know [more]” (R05, M, Chinese, GP Researcher, <1). Secondly, strategies could include updating and engaging existing researchers through continuous professional education, specific workshops and academic conferences: “It could even be in courses where researchers are taught how to do a research proposal and stuff like that” (R09, M, Chinese, GP Researcher, 1‐5).

Participants also highlighted the need to bridge the gap between research and the public, evidenced in a lack of understanding with a direct impact on the willingness of patients and the public to become involved:

I think it's more of public understanding about research and education, whether they want to be involved or not. Sometimes it's like you want them to be involved, but then they're not quite at that level of understanding, so it's difficult to get them to volunteer or donate the time. (R15, Chinese, F, Professor, >10)

## 4 DISCUSSION

This qualitative study suggests that although researchers in Singapore lack experience of PPI, they recognized it as having a potential benefit and identified a number of reasons for adoption. Such reasons were predominantly utilitarian, around practical help, for example, in improving recruitment, retention and increased impact. However, there was also recognition of the symbiotic relationship between researchers and participants, as members of a shared community, and the consequent responsibility of researchers (and funders) to treat people as people and legitimate the position of PPI in research. Yet, in line with other research,^52^, ^53^ this was tempered by a lack of awareness of the expertise that lay perspectives bring to research resulting in evidence of paternalism.^23^ Some researchers in this study, as in other work,^54^, ^55^, ^56^, ^57^, ^58^ struggled to see the value of PPI and were reluctant to share power and control of their work, seeing patients and the public as passive subjects of research. This is reflected in existing literature describing Singapore's hierarchical society,^33^, ^59^ in which lay people rarely challenge the perceived expertise and authority of professionals and researchers.^30^, ^33^ Such power imbalance may be deepened by a poorer understanding of research, lower literacy and poorer English amongst those with the greatest health needs, particularly older populations,^60^ with a consequent reluctance to speak out and/or engage with research.

Other challenges to PPI identified in this study include it being viewed as time‐ and energy‐intensive, and as an added hurdle to developing and carrying out a research project, a concern that is also expressed in the wider literature.^5^, ^61^ In 2015, the NIHR in the UK 62 concluded that despite PPI being progressively adopted, there were “inconsistencies in practice and implementation” and cited negative attitudes of researchers as a major barrier. Consequently, PPI is still often relegated to the role of “thinker at the edges,”^58^, ^63^ within a dominant positivist research paradigm^64^ that hinders the inclusion of “lived experiences” of health and illness.

### 4.1 Possible ways forward

With 85% of health research globally avoidably wasted, in part because of a lack of relevance to patients and the public,^65^, ^66^, ^67^ PPI was seen by researchers in this study to offer opportunities to increase relevance, effectiveness and efficiency. Singapore's ageing population and the concomitant increasing demands on health services were frequently cited as reasons for seeking ways to maximize best use of resources and increase impact. Our findings highlight the ubiquity of social hierarchies in Singapore, an issue recognized as a critical challenge to meaningful PPI.^55^, ^56^

However, it may be that the socio‐political context of Singapore, with its emphasis on Eastern cultural notions of collectivism,^68^ offers opportunities to address this. In a context where society is promoted “above self,”^69^ and in which “to be a citizen is to participate in the civic affairs of the community where one lives, extending outwards to the nation,”^1^ emphasizing PPI as an opportunity to contribute to the wider community good may be an appropriate way forward. This would be in keeping with the World Health Organisation's^70^ emphasis on the centrality of communities in setting their own health priorities and resource allocation.^71^ In Singapore, notwithstanding rapid changes in the composition of the nuclear family, the family remains the primary unit of support, as legislated under the Maintenance of Parents Act (1995), and many people live with other family members. This presents an opportunity to consider ways of implementing PPI at an inter‐generational, household level and within community groups.^72^, ^73^, ^74^ Methods could build on current methodological innovations such as community co‐design^75^ and cultural animation.^28^ In addition to identifying and prioritizing research questions, this participatory collective approach could include participation throughout the research process, including data analysis, dissemination and implementation.^20^, ^28^ It offers a mechanism for traditionally received wisdom to be shared and to contribute to an inclusive dialogue of mutual respect and learning, building trust and potentially leading to enhanced capacity to adapt within what is a rapidly changing landscape of health and health care. There are a number of specific strategies to support PPI that might be considered.

First, as participants mentioned, clarity is needed on the boundaries between PPI and research, including the ethical dimensions. We argue that PPI in research is rooted in ethical principles, that is on the grounds that PPI in research results in increased benefits for all, and that there is a moral requirement to take all possible safeguards to ensure people are not used as a means to an end. Consequently, a clear case for not including PPI in health research should be made at the ethical review stage. Incorporating such a requirement mandates consideration of PPI, raising awareness and contributing to the development of expertise around its implementation.

Also critical to the success of PPI is a strategic approach to education and awareness raising amongst researchers and the public. Such education and awareness raising could take place within a framework that underlines the principles that are central to the culture of Singapore, in particular, the emphasis on the wider community good. Such a strategy could include education across the life course both for researchers and the public, starting at school and undergraduate level and continuing into adult learning arenas covering issues such as why research is important, the importance of diversity, the nature and role of lay and professional expertise and community engagement strategies. Careful consideration of language is fundamental to such a process of awareness raising, not only in the avoidance of jargon but also in developing resources to work with those for whom English is not a first language. Finally, it could include developing culturally appropriate, innovative methods of engagement as a means of ensuring that people feel their contribution is valued regardless of their background or opportunities for formal education.

### 4.2 Strengths and limitations of this study

This is the first study to explore the perspective of those working in health research in Asia on PPI. It identifies the potential for PPI in a non‐Western setting, together with a number of challenges and opportunities that are culturally‐specific. To do this, it drew on a range of views, from those with extensive research experience to those with very little. The majority were Singaporean, and all three Caucasians had lived in Singapore for 10 years or more. Although one individual with little experience did express particularly negative views (R05, M, Chinese, GP Researcher, <1), we found very little difference between the views of those more or less experienced.

A further strength of the study was the contribution of researcher reflexivity. In an attempt to limit the impact of implicit assumptions on the interview process, no participants were working colleagues. The study was undertaken as part of a doctoral research project by LLP, a non‐Singaporean. An initial concern centred on the possibility of researchers giving limited data to someone they might perceive as inexperienced and an outsider. In fact, participants gave very generously, with 13 of the interviews running over time. All of the interviewees commented that she was well positioned to critically evaluate the situation in a way that a Singaporean might not. In addition to detailed use of the contact summary forms and memo writing, this process of reflection also featured in analysis discussions with the other authors (HES and BB). The process informed both data collection (refining the topic guide) and analysis.

A further strength of this research is its contribution to the growing body of evidence calling for a radical re‐thinking of how PPI can be integrated into research in ways that are meaningful and which also maximize its potential impact.^76^, ^77^, ^78^ It also supports the argument for clarification on the relationship between PPI, research and research ethics.

Whilst the study includes a diverse range of participants, in terms of research experience, Singapore is itself unique in Asia. The findings from this study, therefore, require more detailed examination in the context of other Asian cultures. Finally, and most importantly, this study focuses only on the views of health researchers; further work is currently exploring the views of patients and the public.

## 5 CONCLUSION

The socio‐political context of Singapore offers opportunities to translate the traditional individualism prevalent in Western notions of PPI into a more Asian culturally sensitive model of involvement based on family and local community values. Such a participatory collective model offers the prospect of developing sustainable mechanisms for understanding communities more thoroughly and appreciating what they can offer in addressing and adapting to the health challenges facing the nation. Such a model for PPI will still require engagement with many of the complex challenges identified in contexts where PPI is more established, not least addressing understandings and appreciation of the nature of lay knowledge and expertise and its role in research. In the light of growing awareness amongst the wider research community of the need to make PPI more inclusive, such a model would have the potential to contribute to developments elsewhere.

## CONFLICT OF INTEREST

There was no financial support or other benefits from commercial sources for the work reported on in the manuscript, or any other financial interests that any of the authors may have, which could create a potential conflict of interest or the appearance of a conflict of interest with regard to the work.

## DATA AVAILABILITY

This study is not yet complete. Anonymized data will be made available at the university depository in line with best practice when study is completed and data are fully published.
