# Supplementary material for: Researchers' perspectives on public involvement in health research in Singapore: The argument for a community‐based approach
Source: Health Expect. 2019 Jul 19;22(4):666–75. doi: 10.1111/hex.12915 (PMC6737771; doi:10.1111/hex.12915)
Supplement: Supplementary file 1 [file HEX-22-666-s001.docx]

Table S1 Details of six potential participants contacted but not interviewed

| **Gender** | **Ethnic Group** | **Position** | **Reason for rejection** |
| --- | --- | --- | --- |
| Female | Malay | Research Assistant | Cancelled arranged interview: busy |
| Male | Caucasian | Research Fellow | Not working directly with human participants |
| Male | Chinese | GP | Declined: felt they did not have enough research experience |
| Female | Chinese | Research Associate | Cancelled arranged interview: busy |
| Female | Chinese | Professor | Could not find a suitable time |
| Male | Caucasian | Professor | Could not find a suitable time |
